# Photoacoustic Drug Delivery

**DOI:** 10.3390/s17061400

**Published:** 2017-06-15

**Authors:** Yuqi Zhang, Jicheng Yu, Anna R. Kahkoska, Zhen Gu

**Affiliations:** 1 Joint Department of Biomedical Engineering, University of North Carolina at Chapel Hill and North Carolina State University, Raleigh, NC 27695, USA; yzhang96@ncsu.edu (Y.Z.); jyu17@ncsu.edu (J.Y.); 2 Center for Nanotechnology in Drug Delivery and Division of Molecular Pharmaceutics, UNC Eshelman School of Pharmacy, University of North Carolina at Chapel Hill, Chapel Hill, NC 27599, USA; 3 Department of Medicine, University of North Carolina at Chapel Hill, Chapel Hill, NC 27599, USA; anna_kahkoska@med.unc.edu

**Keywords:** photoacoustic imaging, drug delivery, chemotherapy, photothermal therapy

## Abstract

Photoacoustic (PA) technology holds great potential in clinical translation as a new non-invasive bioimaging modality. In contrast to conventional optical imaging, PA imaging (PAI) enables higher resolution imaging with deeper imaging depth. Besides applications for diagnosis, PA has also been extended to theranostic applications. The guidance of PAI facilitates remotely controlled drug delivery. This review focuses on the recent development of PAI-mediated drug delivery systems. We provide an overview of the design of different PAI agents for drug delivery. The challenges and further opportunities regarding PA therapy are also discussed.

## 1. Introduction

**Figure 1:**
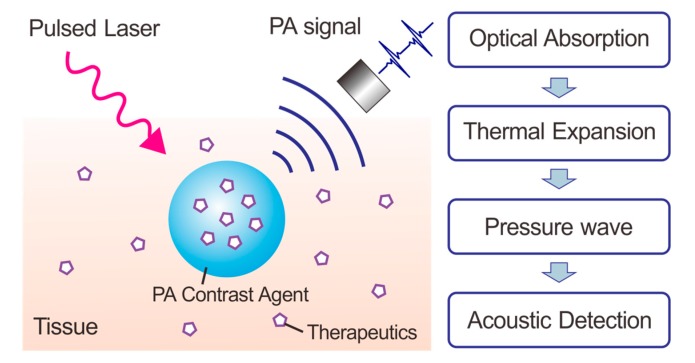
Schematic illustration of photoacoustic imaging and drug delivery.

Photoacoustic (PA) technology has emerged as a promising imaging modality that has attracted increasing interest for biomedical applications during the past decades [1,2,3,4]. The PA effect, first discovered by Alexander G. Bell in 1880 [5,6], describes the formation of acoustic waves following absorption of light pulses. More than a century after the discovery of the PA effect, photoacoustic imaging (PAI) was introduced to biomedical applications. Since the pioneering work by Wang et al. that realized in vivo PAI in a rat brain in 2003 [7], non-invasive PAI has been extensively explored as a technology for biomedical imaging and diagnostics [8,9,10,11,12,13]. The mechanism of PAI is based on the energy transformation among light, heat and sound (Figure 1). When illuminated with a pulsed laser, PAI contrast agents in tissue rapidly absorb energy and generate heat causing thermalelastic expansion and emitting mechanical pressure waves at ultrasonic frequencies. The periodic sound waves can be detected by pressure transducers close to the tissue and output to form images. This imaging mechanism endows deeper tissue penetration up to 5–6 cm as compared to the traditional optical imaging modalities that have limited imaging depth within 1 mm [14]. In addition, PAI imaging offers higher spatial resolution up to 5 μm, due to the weaker scattering of ultrasonic signals as compared to light signals [15,16,17,18].

Certain endogenous molecules, such as hemoglobin and melanin, can function as PAI agents due to the inherent optical absorption of these molecules [2]. Exogenous imaging agents with high sensitivity and specificity have been widely exploited to further enhance the imaging signal, especially those materials with strong absorption in the near-infrared (NIR) region where optical attenuation is weaker than the visible light region in vivo. In addition to endogenous molecules, many optical nanomaterials exhibit potential as PAI agents, including metallic nanoclusters, carbon-based nanostructures, organic dyes, and conducting polymeric nanoparticles [17,19,20,21]. To date, PAI technology has demonstrated great potential in preclinical and clinical diagnosis of cancer [22,23,24,25], detection of tumor metastases [26,27], endoscopic gastrointestinal imaging [28], and monitoring of treatments [29].

Recently, the application of PAI has been extended to imaging-guided photothermal and chemotherapy. The PAI contrast agents that have strong absorbance in the NIR region can usually produce heat under laser irradiation, leading to thermal ablation and subsequent death of targeted cells, which is also termed photothermal therapy (PTT) [30]. Numerous agents have been developed to effectively increase the local temperature from the normal body temperature (37 °C) to over 41 °C to cause hyperthermia treatments, including protein denaturation and cell inactivation; or even up to 48 °C, leading to irreversible thermal damage [31]. The generated local hyperthermia can further promote encapsulated therapeutics diffusion from the agents, as well as facilitate cellular uptake as a result of increased membrane permeability for enhanced chemotherapy [32,33]. It was reported that the ultrasonic pressure produced by PA effect could be used to promote transdermal drug delivery, as well as localized drug release in vessels [34,35,36]. Researchers have also explored PAI agents as drug carriers, where the heat generated by the agent upon laser irradiation can control the drug release in an on-demand manner. In this review, we summarize the latest advances in PAI-assistant therapy and discuss representative examples of different material-based drug delivery systems.

## 2. Inorganic Nanomaterials-Based Photoacoustic Therapy

Among the inorganic materials that have been explored, metal nanomaterials such as noble metal (Au, Pd) nanoparticles [37,38,39,40] and transition metal dichalcogenides (WS_2_, TiS_2_, WO_x_, Cu_2−x_Se) [41,42,43,44,45,46,47,48] have attracted a great deal of attention in PAI application, owing to their desirable optical, chemical and biological properties. Another typical class of PAI agents is carbon-based nanomaterials, which include carbon nanotube and reduced graphene oxide [49,50,51,52]; and these materials possess strong optical absorption. In addition, this class has been investigated as drug carriers via functionalization or integration with other materials for PAI guided therapy [52]. The following section provides an overview of the most recent development of inorganic materials for use as both PAI contrast agents and therapeutic carriers.

### 2.1. Metallic Nanomaterials

Gold-based (Au) nanostructures comprise another dominant class of PAI contrast agents. Gold nanostructures, such as nanorods (NRs) [44,53,54,55], nanocages (NCs) [37,56,57,58], and nanospheres (NSs) [59], have strong and tunable optical absorption due to localized surface plasmon resonance (LSPR) effect, where the free charges on the surface of the Au nanostructures oscillate with the electromagnetic field. Additionally, the absorption region can be adjusted as a result of change in resonance frequency by modulating the size and shape of the Au nanostructures [60].

**Figure 2:**
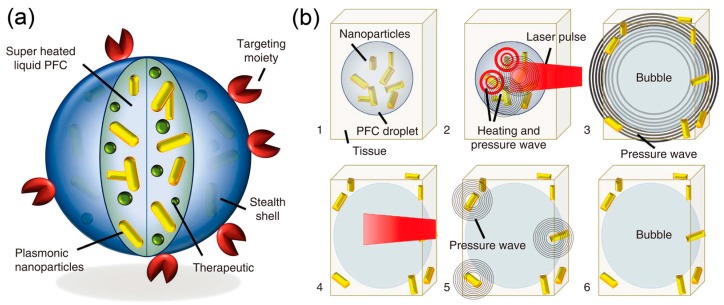
Schematic of Au NRs and therapeutics loading perfluorocarbon nanodroplet as PAI contrast agent. (**a**) Diagram showing structure of the PA nanodroplet; (**b**) Illustrations indicating the mechanism of PA activation of nanodroplet. @2011 Nature Publish Group [64].

Numerous reports have demonstrated that Au nanostructures may be designed as drug carriers for PAI-guided chemo/photothermal therapy (PTT) [20,21,37,61,62,63]. For example, Wilson et al. developed liquid perfluorocarbon nanodroplet-encapsulated Au NRs as a PA and ultrasound imaging agent, as well as a potential drug carrier (Figure 2) [64]. The heat generated by Au NRs under laser causes the perfluorocarbon to undergo a phase transition (liquid-to-gas), resulting in subsequent loading release. The researchers found that the PA signal was enhanced due to the vaporization of perfluorocarbon as compared to the signal from Au NRs that were produced thermal expansion only. Taking advantage of this design, the Xing group loaded paclitaxel and Au NRs into perfluorohexane nanodroplets for PAI-guided chemotherapy and additionally functionalized the nanodroplets with folic acid for tumor targeting [65]. Upon application of a pulsed laser, the nanodroplets were rapidly destroyed due to the vaporization of perfluorohexane, resulting in rapid drug release. Researchers have demonstrated enhanced tumor suppression as a result of photoacoustic-chemo synergistic treatment, where the integration of chemotherapy with PTT not only achieved controlled and localized drug release, but also promoted the cellular uptake, thus leading to improved therapeutic efficacy. Moreover, the chemotherapy was shown to suppress tumor recurrence and metastasis that may be caused by inhomogeneous ablation with PTT alone.

**Figure 3:**
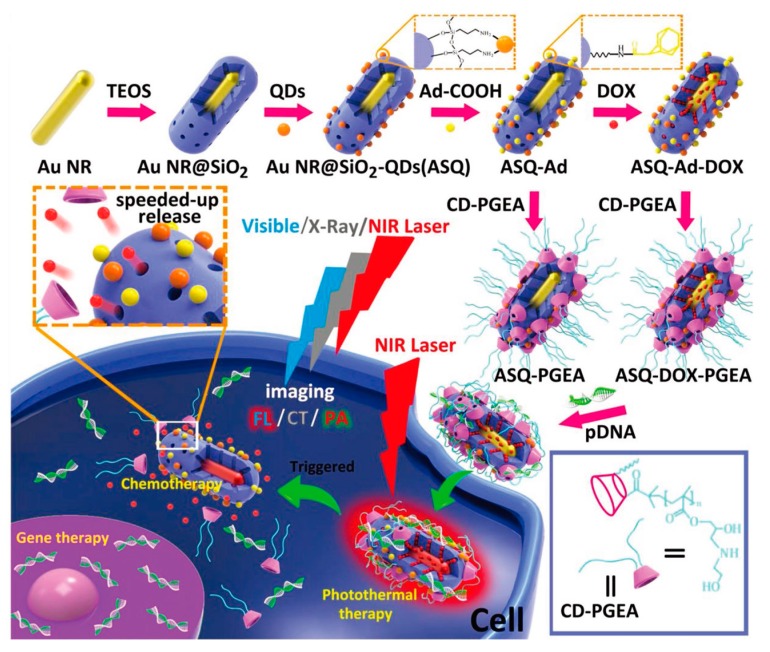
Schematic illustration of PAI-guided gene/chemo/photothermal triple therapy based on mesoporous silica coated Au NRs. @2017 Wiley [66].

Duan et al. designed a multimodal imaging-guided combination therapy system based on Au NRs [66]. The researchers first coated the Au NRs with mesoporous silica for loading of drug and quantum dots, and further decorated the system with adamantyl (Ad) groups and two-armed ethanolamine-modified poly(glycidyl methacrylate) with cyclodextrin cores (CD–PGEA) conjugates as gatekeeper (Figure 3). Additionally, pDNA was condensed to the surface of the nanostructure for gene therapy. The obtained ASQ-DOX-PGEA2-p53 (Au NRs@SiO_2_-QDs (quantum dots)/DOX (doxorubicin)-Ad/CD-PGEA2/p53) nanoplatform was designed to serve as modality for fluorescent imaging, CT imaging, as well as PAI, with the additional advantageous photothermal properties to effectively inhibit glioma tumor growth with application of NIR. Furthermore, the heat generated by Au NRs under NIR irradiation reduced the supramolecular interactions between Au and CD-PGEA, thereby opening the pore gate and leading to subsequent release of pDNA and DOX encapsulated in the mesoporous silica layer. Such multifunctional nanoparticles, integrating An NRs, silica layers, QDs, and CD-PGEA as one structure, leveraged the advantages of each component to realize precise imaging-guided gene/chemo/photothermal triple-combination therapy of cancer. Besides coating Au NRs with mesoporous silica, Huang et al. designed a “yolk-shell” structure, incorporating a “non-contact” porous magnetic nanoshell on Au NRs functioning as both the drug container and magnetic resonance imaging (MRI) agent [67]. Under the guidance of PAI and MRI, the drug carriers were magnetically guided to the tumor site with on-demand drug release triggered by pH/NIR. The synergistic chemo-photothermal therapy was shown to be effective in eradiating and suppressing recurrence of 4T1 tumor in mice.

Alternatively, Lee et al. employed hollow Au nanopheres (HAuNSs) as both the drug carrier and PAI agent [68]. The prepared DOX@ PEG (poly(ethylene glycol))-HAuNSs was shown to initiate the release of DOX upon application of a 3-W NIR laser. In vivo experiments with PAI at tumor site also revealed a substantial increase in temperature from 37 °C to more than 50 °C with laser irradiation, thereby inducing effective photothermal ablation to inhibit tumor growth. Xia group designed a hybrid system for bioimaging and therapy by integrating Au NCs with phase-change material (PCM) [69]. These Au NCs were shown to function as a contrast agent for enhanced PAI, while the PCM, 1-tetradecanol, acted as the drug reservoir for controlled release in response to heat. When the local temperatures surpassed the melting point of the PCM, drug was able to diffuse through Au NCs.

Cai et al. employed hollow mesoporous Prussian blue nanoparticles (HMPBs) as a PAI contrast agent [70]. Prussian blue, which is widely used in clinical medicine, has strong optical absorbance in NIR region owing to the charge transition between Fe(II) and Fe(III). Recently, Prussian blue has attracted increasing interest for use in PAI and PTT [71,72,73,74]. In this theranostic work, the researchers embedded perfluoropentane and DOX in HMPBs to form a versatile platform that could deliver DOX under the guidance of PA/ultrasound dual mode imaging and realize on-demand chemo/thermal therapy. To enhance the blood circulation time of HMPBs for improved anticancer efficacy, Chen et al. camouflaged the HMPBs by coating the system with an additional layer of the red blood cell membrane [75]. The red blood cell membrane on the surface was shown to effectively protect the HMPBs from body clearance. After 24 h-injection, 17.5% per injected dose per gram of tissue (ID/g) of coated HMPBs were still maintained in blood circulation, in contrast to the 6% of bare HMPBs remaining in circulation at the same time point. In addition, the coating of red blood cell membrane had the advantage of minimizing immune response.

In addition to noble metallic nanoparticles, transition metal based nanomaterials also exhibit intriguing PA properties. Oxygen deficient molybdenum oxide (MoO_3-x_) nanocrystals are one of the typical nonstoichiometric metal chalcogenides, which possess significant LSPR absorption in the NIR region via certain specific synthesis process [76]. Bao et al. employed poly(ethylene glycol) (PEG) functionalized MoO_3−x_ hollow nanospheres (PEG-MoO_3−x_ HNSs) as a PAI agent for imaging-guided chemo-photothermal therapy [77]. The PEG not only functioned as a stabilizer to prevent MoO_3−x_ HNSs from aggregation, but also shifted the LSPR absorption peak from visible to NIR range due to its reduction function. Camptothecin, an insoluble anticancer drug, was then encapsulated in the nanosphere, where its release kinetics was shown to be effectively controlled by pH and NIR. The in vivo study showed remarkable synergism in the anticancer effect inpancreatic tumor-bearing mice. Song et al. have also reported cobalt chalcogenides materials as the theranostic agent, achieved by acrylic acid conjugated Co_9_Se_8_ nanoplate of 100 nm in diameter and 6 nm in height [46]. With 10 min laser exposure (808 nm, 1 W/cm^2^), the PAA-Co_9_Se_8_ solution (30 μg/mL) increased the temperature by 26 °C. DOX was further loaded to the nanoplates via hydrophobic interactions with a pH-dependent and laser-triggered release dynamics for chemotherapy. The nanoplates exhibited strong NIR absorbance and low toxicity, suggesting promise for this system as a PAI contrast agent as well as a candidate for PTT.

### 2.2. Carbon-Based Nanostructures

Carbon nanotube exhibits strong optical absorbance from visible to NIR regions, making it a robust PAI contrast agent [50,51,78,79]. It is also able to produce effective heat for photothermal ablation [80,81,82].

**Figure 4:**
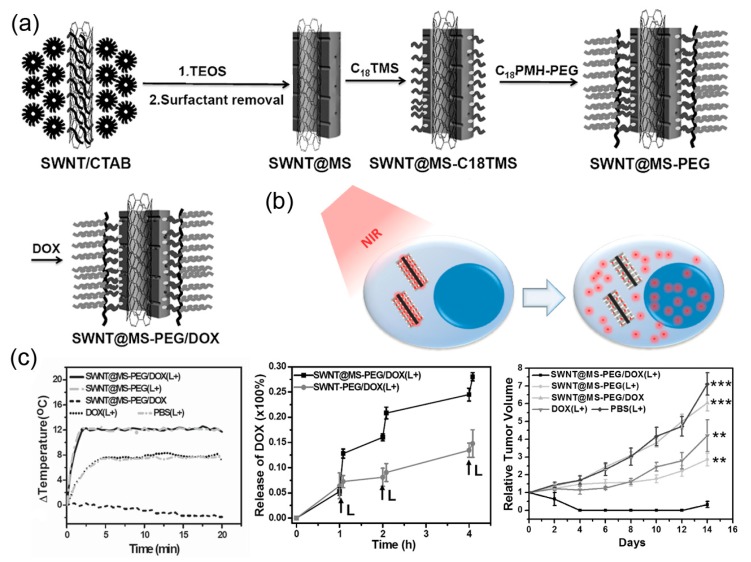
Schematic of PAI-guided chemo/photothermal therapy based on mesoporous silica coated SWNTs. (**a**) Diagram showing preparation of DOX loading mesoporous silica modified SWNT; (**b**) A scheme of NIR-triggered intracellular DOX release from SWNT; (**c**) Temperature change curves of tumors with SWNT@MS-PEG under 808 nm laser irradiation of 0.5 W/cm^2^ for 20 min showing effective heat generation (**left**); The NIR-triggered DOX release profiles (**middle**); Tumor growth curves of mice with different treatment indicating synergistic tumor inhibition effect by chemo/photothermal therapy using SWNT@MS/DOX(Laser+) (**right**). @2015 Wiley [84].

In addition, carbon nanotubes can also be used to load and deliver therapeutics for chemotherapy [83]. To this end, Liu and coworkers exploited a NIR-triggered drug delivery system based on mesoporous silica coated single wall carbon nanotube (SWNT) for diagnosis and therapy of tumors (Figure 4), demonstrating accumulation of drug carriers in tumor via PAI and MRI [84]. 

The SWNT was coated with mesoporous silica (MS) for drug loading, and further PEG function on the surface for protection from fast body clearance (Figure 4a). The SWNT rapidly increased the tumor surface temperature to ~48 °C and maintained during the laser irradiation (808 nm, 0.5 W/cm^2^ for 20 min) as monitored by an infrared thermal camera (Figure 4c). The local hyperthermia generated by SWNT under NIR promoted rapid therapeutic release, resulting in a synergistic cancer cell killing efficacy by the integration of chemo and photothermal therapy (Figure 4b). As presented in the tumor growth curves (Figure 4c), the PTT (SWNT@MS-PEG(L+) group) could inhibit tumor growth partially, even better than the chemotherapy (DOX group), contributing to the effective photothemal effect on cell killing. However, the SWNT@MS-PEG/DOX(L+) group showed remarkably enhanced tumor suppression effect than PTT only as a result of synergistic therapy.

Reduced graphene oxide (rGO) can also absorb light, while it has relatively low photothermal conversion efficiency. Its optical properties can be improved via integration with other plasmonic materials [48,85,86,87,88]. Chen and Ma group reported enhanced photothermal effect via coating a plasmonic Au shell on rGO [89]. The Au nanorod vesicle (NRVe) was self-assembled by amphiphilic PEG-grafted Au NRs, which functioned as a cavity that concentrated the electromagnetic radiations and resulted in raised photo absorption. Anticancer drug DOX was then loaded on rGO by *π*-*π* stacking interaction. With a relatively low laser irradiation (808 nm, 0.25 W/cm^2^), the rGO-Au NRVe-DOX produced effective heat at the tumor site, promoting targeted drug release to tumor cells.

## 3. Organic Nanomaterials-Based Photoacoustic Therapy

In the realm of organic materials, small molecule dyes and semiconducting polymeric nanoparticles have recently emerged as a new class of PAI agents [90,91,92,93,94,95,96,97]. These materials have been extensively developed and also applied on theranostic platforms [98,99,100,101,102,103,104].

For example, Liu group encapsulated an NIR dye IR825, and a photosensitizer (chlorin e6) in nanomicelles as PAI agent for imaging guided PTT and photodynamic therapy (PDT) [105]. The NIR dye accumulated at tumor sites absorbed photon energy and converted it to heat, inducing a photothermal effect to inhibit tumor expression. The photosensitizer enabled transformation of photo energy to the surrounding oxygen molecules, generating reactive oxygen species, such as singlet oxygen (^1^O_2_), to kill cells, which is named PDT. Wang et al. designed a pH-responsive micelle comprised of a pH-sensitive diblock polymer, the photosensitizer chlorin e6, and a pluronic prodrug of DOX [106]. Under acidic conditions (pH < 6.3), the photoactivity was activated and was shown to generate reactive oxygen species under NIR exposure to initiate drug release and PDT. The micelle also transferred light to heat, thereby inducing local hyperthermia for PTT as well as functioning as a PAI contrast agent. Cai et al. designed an organic PAI agent via self-assembly of a donor-acceptor-donor (D-A-D) small molecule base on diketopyrrolopyrrole-triphenylamine [107]. The formation of particles enhanced the D-A-D structure, which promoted charge transport capacity and generated heat upon laser irradiation (660 nm, 1 W/cm^2^), as was shown to be efficient for tumor inhibition as a result of PTT/PDT synergistic therapy. Recently, Zhang et al. synthesized an electron donor-acceptor (D-A) conjugated polymer for light absorption [108]. They further introduced a light-harvesting unit to the side chain in order to enhance photothermal conversion. The polymeric nanoparticles prepared using nanoprecipitation methods exhibited effective PAI-guided PTT, reaching a photothermal conversion efficiency of 62.3%. Taken together, these studies illuminate the potential of small organic nanoparticles for PAI-guided therapy.

The Cheng group took advantage of an endogenous PAI agent, melanin, to fabricate PA nanoparticles [109]. Melanin is a natural biopolymer that exists in many organisms and possesses intrinsic PA properties in addition to ideal properties for molecular imaging, including good biocompatibility and biodegradability [110]. The researchers synthesized ultra-small melanin nanoparticles (MNPs) with a size of around 7 nm, which can be utilized for PAI due to its native optical properties. In addition, the resulting MNPs were shown to actively chelate to metal ions such as Cu^2+^, Fe^3+^ for magnetic resonance imaging (MRI) and positron emission tomography (PET). They further loaded a hydrophobic drug, sorafenib (SRF), on PEG-functionalized MNPs via π–π interactions and formed a larger MNPs aggregate with a size of 60 nm for imaging-guided chemotherapy [111]. Subsequent in vivo studies indicated that the SRF-MNPs successfully transported drug into tumor via intravenous injection and required much lower dose (4 mg kg^−1^) as compared to oral administration (20 mg kg^−1^).

**Figure 5:**
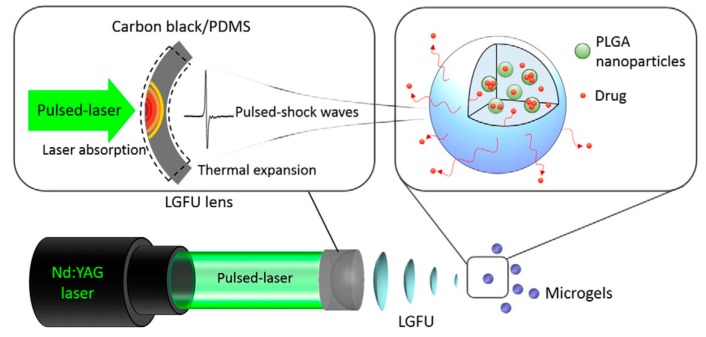
Schematic of laser-generated-focused ultrasound triggered drug delivery system. @2015 Elsevier [36].

With the assistance of a transducer that comprised of a laser absorption layer (carbon nanotube) and a thermal expansion layer (polydimethylsiloxane), pulsed laser can be transmitted to high frequency ultrasound [112]. The Gu and Jiang groups utilized laser-generated-focused ultrasound (LGFU) to trigger drug release from the microgel (Figure 5) [36]. Compared to traditional ultrasound triggered drug delivery systems, LGFU showed higher resolution and reduced heating effect to normal tissue. Anticancer drug DOX and antibacterial drug ciprofloxacin were encapsulated in PLGA nanoparticles, after which the PLGA nanoparticles were further embedded into alginate microgel. A significantly enhanced release of drug was observed at the focal spot after application of LGFU by the cavitation effect. The researchers further validated that this LGFU-triggered delivery device could be leveraged to provide spatiotemporally controlled release for in vitro antitumor and antibacterial treatment.

## 4. Conclusions

In summary, PAI technology has been developed rapidly in the past decades and continues to see substantial expansion of capabilities and refinement of technique. Furthermore, PAI-assisted drug delivery systems have displayed promising evidence of synergistic treatment efficacy. However, such systems still face multiple challenges in terms of clinical translation. For instance, although metallic nanoparticles exhibit excellent and tunable optical properties, there are concerns with regards to their systemic cytotoxicity in long-term treatment. Therefore, further investigations into biocompatibility are critically needed. Although small molecular dyes offer better biocompatibility and relatively low cost-two benefits which have conferred wide application of these systems in biomedicine-poor photostability and short retention time are two limitations that need to be addressed prior to translational studies. Semiconducting polymer particles have emerged as alternative PAI agents with enhanced stability [113], yet their relatively complicated and costly preparation process represents a barrier for further translation. The study of improvement upon these limitations will ultimately allow PA technology to move forward as a competitive non-invasive bioimaging and treatment modality in clinic [114,115].
